# Perilesional Diffusion Tensor Imaging for Differentiating Malignant and Benign Causes of Intracerebral Hemorrhage

**DOI:** 10.3390/diagnostics16132054

**Published:** 2026-06-30

**Authors:** Mehmet Fatih Erbay, Ahmet Turan Kaya, İsmail Okan Yıldırım

**Affiliations:** Department of Radiology, Turgut Özal Medical Center, Faculty of Medicine, Inonu University, 44280 Malatya, Türkiye; hmttrnky.62@gmail.com (A.T.K.); ioyildirim@gmail.com (İ.O.Y.)

**Keywords:** diffusion tensor imaging, intracerebral hemorrhage, perilesional edema, diffusivity metrics

## Abstract

**Background/Objectives**: To evaluate the discriminative value of perilesional microstructural alterations assessed by diffusion tensor imaging (DTI) parameters in differentiating malignant and benign causes of intracerebral hemorrhage. **Methods**: This retrospective study included patients with intracerebral hemorrhage classified as benign or malignant based on follow-up findings and histopathological diagnosis. Fractional anisotropy (FA), axial diffusivity (AD), radial diffusivity (RD), mean diffusivity (MD), lesion-to-normal (L/N) ratios, and perilesional edema index (PEI) were extracted from standardized perilesional regions. Diffusion parameters were compared between groups, and their discriminative performance was assessed using ROC analysis and logistic regression. **Results**: A total of 41 patients (20 benign, 21 malignant) were included. Malignant cases demonstrated significantly greater PEI and distinct diffusion abnormalities, with markedly higher AD, RD, and MD values, as well as elevated L/N ratios (all *p* < 0.001). FA was significantly reduced in malignant cases (*p* = 0.016). **Conclusions**: Diffusivity metrics—particularly AD, RD, and MD values and their L/N ratios—demonstrated excellent diagnostic performance in differentiating malignant from benign causes of intracerebral hemorrhage, further informed by the PEI, and may support clinical decision-making in diagnostically challenging cases.

## 1. Introduction

Intracerebral hemorrhage (ICH) is a serious, life-threatening condition associated with high morbidity and mortality worldwide. Depending on the underlying etiology, ICH is commonly categorized as primary or secondary [1,2]. Notably, bleeding may be the initial clinical presentation of both primary and metastatic brain tumors and can obscure the underlying pathology, frequently leading to misdiagnosis as non-neoplastic ICH [3,4]. Conversely, certain non-neoplastic conditions, including subacute arterial ischemia, venous infarction, or herpes encephalitis, may be misinterpreted as hemorrhagic malignant tumors due to associated mass effect, contrast enhancement, and the presence of blood products [5]. Therefore, accurate etiological differentiation of ICH on conventional imaging remains challenging at initial presentation, as tumor-related hemorrhages may mimic both primary and secondary ICH [6,7,8]. Early recognition of the underlying etiology is essential for guiding appropriate management, enabling timely surgical intervention when needed while avoiding unnecessary procedures in lesions suitable for conservative treatment or imaging follow-up [3]. Although non-contrast CT is the first-line modality for the initial assessment of ICH, intravenous contrast administration is often required to investigate a potential underlying tumoral component. However, solid tumor enhancement on CT may be obscured by the high attenuation of acute blood products, and similarly, the variable T1 signal characteristics of hematomas on MRI can mask tumoral enhancement in the acute or subacute stage of bleeding [2]. This diagnostic challenge underscores the need for advanced imaging biomarkers, which was the focus of the present study. Several advanced imaging techniques have been proposed to improve etiological assessment of ICH; however, accurate characterization often requires a multimodal imaging approach, particularly in the acute setting [9].

Importantly, perilesional edema of a malignant tumor does not necessarily represent a purely vasogenic process but may also contain infiltrating tumor cells and other reactive elements, further contributing to diagnostic ambiguity on standard imaging modalities [10]. In this context, DTI enables in vivo assessment of tissue microstructure through directional diffusion and offers complementary perilesional information less influenced by hemorrhagic signal effects [11].

Despite studies investigating the role of various imaging techniques, including dual-energy CT (DECT), CT densitometry, CT angiography, and MR spectroscopy, in differentiating malignant and benign hemorrhagic brain lesions [2,3,12,13], to the best of our knowledge, no studies have specifically evaluated the diagnostic utility of perilesional microstructural changes using DTI for this purpose. Therefore, in the present study we aimed to investigate whether quantitative DTI parameters obtained from the perilesional region, in conjunction with perilesional edema characteristics, can improve differentiation between malignant and benign hemorrhagic brain lesions.

## 2. Materials and Methods

### 2.1. Study Design and Patient Selection

This retrospective study was conducted at a single tertiary referral center. Patients with hemorrhagic brain lesions who underwent a contrast-enhanced brain MRI protocol including a DTI sequence between 2018 and 2024 were screened from offline hospital records. Consecutive patients meeting the predefined inclusion criteria during the study period were retrospectively included. Patients were categorized into benign and malignant groups. Malignant lesions were confirmed histopathologically, whereas lesions demonstrating regression or complete resolution on clinico-radiological follow-up imaging, typically obtained within 3–6 months, were classified as benign. Patients with confirmed intraparenchymal hemorrhage on brain MRI who had either definitive histopathological diagnosis or available follow-up imaging data in the picture archiving and communication system (PACS) were included in the study. Exclusion criteria included prior surgical intervention or radiation therapy at the lesion site, as well as poor image quality due to motion artifacts or other technical limitations. The malignant group consisted predominantly of primary brain tumors (*n* = 17), while a smaller subset comprised metastatic lesions (*n* = 4). The study protocol was approved by the Ethics Committee of Inonu University School of Medicine (IRB authorization number: 2025/7329), and the requirement for written informed consent was waived due to the retrospective study design. The study was conducted in accordance with the Declaration of Helsinki.

### 2.2. MRI Acquisition Protocol and Post-Processing

All MRI examinations were performed on clinically available 1.5 Tesla (Siemens Avanto, Siemens Healthineers, Erlangen, Germany) and 3 Tesla (Siemens Skyra, Siemens Healthineers, Erlangen, Germany) MR scanners using standard head coils. The routine imaging protocol included axial T1-weighted, T2-weighted, FLAIR, DWI, SWI, DTI, and contrast-enhanced sagittal T1-weighted sequences. DTI was acquired using a single-shot echo-planar imaging (EPI) sequence. Due to the retrospective nature of the study, DTI parameters varied slightly according to scanner field strength. Detailed acquisition parameters are summarized in Table 1.

To reduce the potential impact of inter-scanner variability, DTI analyses were based on L/N ratios derived from contralateral mirror-region measurements.

The MRI datasets were subsequently transferred to a dedicated workstation (syngo.via version 10.05.0000.0000, Siemens Healthineers, Erlangen, Germany), where post-processing and quantitative DTI analyses were performed. Post-processing included diffusion tensor fitting, generation of parametric maps, and ROI-based quantitative measurements following automatic correction for motion and eddy current distortions. Parametric maps were co-registered with anatomical sequences, particularly FLAIR images, to ensure anatomically accurate ROI placement within the perilesional edema. Quantitative DTI-derived parameters, including fractional anisotropy (FA), axial diffusivity (AD), radial diffusivity (RD), and mean diffusivity (MD), were extracted from the parametric maps. Mean diffusivity (MD) was not directly provided by the workstation and was therefore calculated using the formula MD = (AD + 2 × RD)/3, where AD corresponds to the principal eigenvalue (λ1) and RD corresponds to the average of the second and third eigenvalues ((λ2 + λ3)/2). Lesion-to-normal (L/N) ratios were calculated for all parameters.

### 2.3. Lesion Identification and ROI Placement

**Figure 1:**
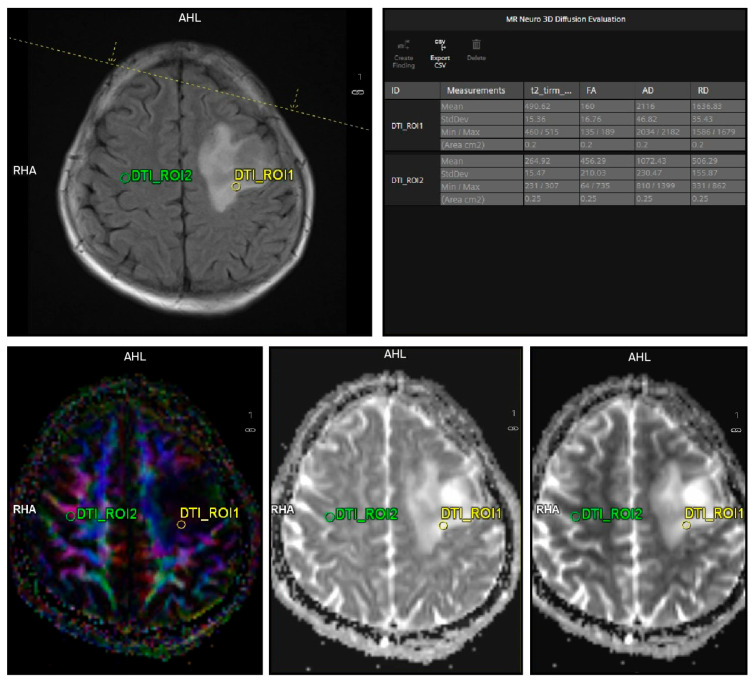
ROI placement strategy for perilesional DTI analysis. ROIs were placed within the immediate perilesional edema adjacent to the lesion margin (DTI_ROI1) and mirrored to the contralateral normal-appearing white matter (DTI_ROI2). Corresponding FA, AD, and RD values were extracted.

Hemorrhagic lesions were identified primarily on SWI and T1-weighted images. The perilesional region was defined as the brain parenchyma immediately surrounding the hemorrhagic core. Perilesional edema was identified on T2-weighted FLAIR images. DTI analysis was performed using a region-of-interest (ROI)-based approach. For ROI placement, the immediate perilesional edema zone adjacent to the lesion margin (as visualized on FLAIR), where edema signal was most prominent, was selected. ROI size ranged between 0.1 and 0.3 cm^2^. For each lesion, a mirror ROI of identical size and shape was placed in the contralateral normal-appearing white matter (Figure 1). Areas of necrosis, cystic components, visible vessels, cerebrospinal fluid spaces, as well as regions showing gliosis or chronic ischemia-related hyperintensities were deliberately excluded from ROI placement. Measurements were performed by experienced neuroradiologists blinded to diagnosis, and care was taken to ensure consistent ROI placement across cases.

### 2.4. Statistical Analysis

All statistical analysis were performed using IBM SPSS Statistics for Windows, version 25.0 (IBM Corp., Armonk, NY, USA). The normality of continuous variables was assessed using the Shapiro–Wilk test. Normally distributed variables were expressed as mean ± standard deviation (SD) and compared between groups using the independent-samples *t*-test, whereas non-normally distributed variables were expressed as median (interquartile range, IQR) and compared using the Mann–Whitney U test. Categorical variables were expressed as frequencies (percentages) and analyzed using the continuity-corrected χ^2^ test.

The receiver operating characteristic (ROC) curve analysis was employed to evaluate the diagnostic performance of diffusion-weighted imaging (DWI) parameters, including fractional anisotropy (FA), axial diffusivity (AD), radial diffusivity (RD), and mean diffusivity (MD). For each parameter, the area under the curve (AUC) with 95% confidence intervals (CI), sensitivity, specificity, positive predictive value (PPV), negative predictive value (NPV), and overall accuracy were calculated. The optimal cut-off values for each DWI metric were determined using the Youden index (J = sensitivity + specificity − 1) to maximize the balance between sensitivity and specificity. Logistic regression analyses were used to determine independent predictors of malignancy. First, univariate logistic regression identified candidate parameters, followed by multivariate models incorporating demographic (age, sex), clinical (perilesional edema index [PEI]) and imaging variables. Each model was evaluated both for continuous parameters and for dichotomized variables based on ROC-derived cut-offs. Odds ratios (ORs) with corresponding 95% CIs and Wald statistics were reported. For ratio-based analysis, lesion-to-normal (L/N) ratios were computed by dividing the lesion-side DTI parameter by its contralateral counterpart. These normalized ratios were subjected to parallel regression and ROC analysis to minimize individual variability and highlight microstructural asymmetry. All *p* values were two-sided, and results with *p* < 0.05 were considered statistically significant throughout the analysis. Interobserver agreement between the two independent radiologists for all quantitative DTI measurements (lesional and contralateral normal-appearing FA, AD, RD, and MD values, together with their L/N ratios) was assessed using the intraclass correlation coefficient (ICC), based on a two-way random-effects model for single measurements with absolute agreement [ICC (2, 1)]. Coefficients were reported with 95% confidence intervals and interpreted according to Koo and Li, with values below 0.50 indicating poor, 0.50–0.75 moderate, 0.75–0.90 good, and above 0.90 excellent reliability [14].

To assess the potential influence of inter-scanner heterogeneity, the distribution of patients across the 1.5 T and 3 T systems was compared by lesion nature using the χ^2^ test, and all diffusion parameters were compared between the two field-strength subgroups using the Mann–Whitney U test. A sensitivity analysis repeating the benign-versus-malignant comparison within each scanner subgroup was performed, and magnetic field strength was added as an additional covariate to each multivariable logistic-regression model to confirm that it did not act as a confounder.

## 3. Results

### 3.1. Interobserver Agreement

Interobserver reproducibility of the measurements was assessed prior to the diagnostic analysis (Table 2). For the lesional metrics, agreement was good for mean diffusivity (ICC 0.808, 95% CI 0.67–0.89) and moderate-to-good for AD and RD (ICC 0.745 and 0.746, respectively), whereas FA showed moderate agreement (ICC 0.612, 95% CI 0.38–0.77). The AD and MD L/N ratios—which provided the strongest diagnostic performance—also demonstrated good agreement (ICC 0.791 and 0.790). Agreement for the contralateral normal-appearing parenchyma was comparatively lower (ICC 0.434–0.653), which is expected given the narrow physiological range of normal-tissue values rather than any inconsistency in measurement. All intraclass correlation coefficients were statistically significant (*p* < 0.001), confirming the reproducibility of the diffusion measurements used in the subsequent analyses.

### 3.2. Patient Characteristics and Perilesional Edema

A total of 41 patients with hemorrhagic brain lesions were included, comprising 20 benign and 21 malignant lesions. There were no significant differences between groups in terms of age (*p* = 0.368), sex distribution (*p* = 0.873), or lesion lateralization (*p* > 0.999). The perilesional edema index (PEI) was significantly higher in malignant lesions (median [IQR]: 80.6 [37.5–160.2] vs. 34.6 [21.5–71.0], *p* = 0.023) (Table 3).

### 3.3. Anatomical and Diagnostic Distribution

Malignant lesions were most frequently located in the frontal lobe (47.6%) and consisted predominantly of primary high-grade gliomas (81.0%) and solitary metastases (19.0%). Benign lesions were more evenly distributed and mainly included venous ischemic changes (45.0%), cavernomas (20.0%), and arterial ischemic lesions (15.0%) (Supplementary Table S1).

### 3.4. DTI Metrics

**Figure 2:**
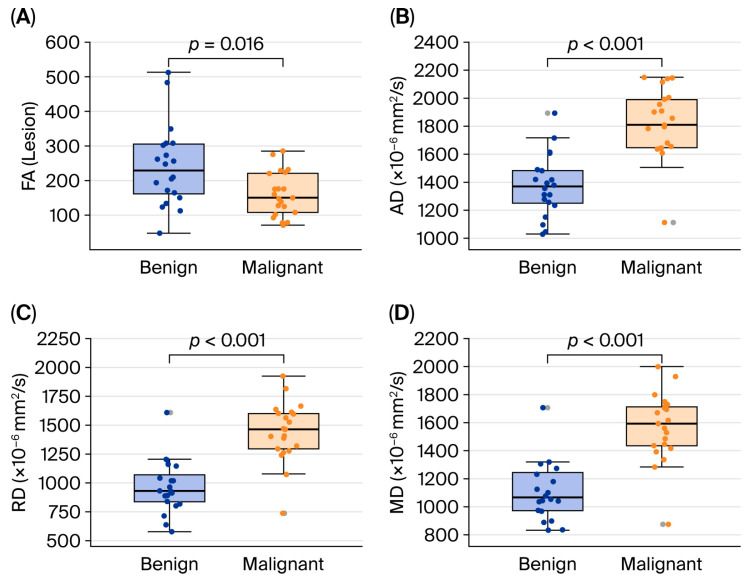
Comparison of absolute lesional DTI parameters between benign (*n* = 20) and malignant (*n* = 21) brain lesions. Box plots show the median (central line), interquartile range (box), and 1.5× IQR whiskers, with individual data points overlaid. (**A**) Fractional anisotropy (FA) was significantly lower in malignant lesions, whereas (**B**) axial diffusivity (AD), (**C**) radial diffusivity (RD), and (**D**) mean diffusivity (MD) were significantly higher in malignant lesions. Between-group differences were assessed using the Mann–Whitney U test.

**Figure 3:**
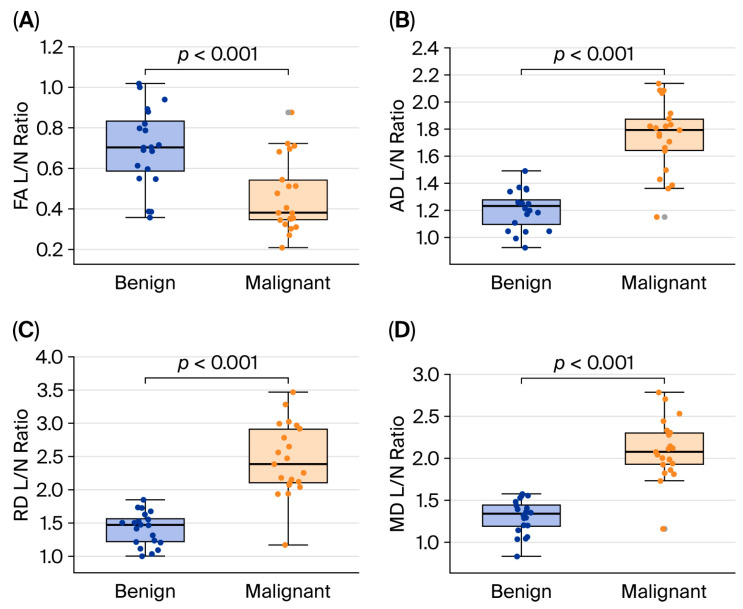
Comparison of lesion-to-normal (L/N) diffusion ratios between benign (*n* = 20) and malignant (*n* = 21) brain lesions. (**A**) The FA L/N ratio was significantly lower in malignant lesions, while (**B**) AD, (**C**) RD, and (**D**) MD L/N ratios were significantly higher, with near-complete separation between the two groups. Box plots show the median, interquartile range, and 1.5× IQR whiskers with individual data points overlaid. Between-group differences were assessed using the Mann–Whitney U test.

Quantitative DTI analysis demonstrated marked differences between malignant and benign hemorrhagic lesions. Malignant lesions showed significantly higher AD, RD, and MD values (all *p* < 0.001), along with lower FA (*p* = 0.016). When normalized to contralateral parenchyma, L/N ratios provided stronger separation: AD, RD, and MD L/N ratios were significantly higher and FA L/N ratios were significantly lower in malignant lesions compared with benign lesions (all *p* < 0.001) (Figure 2 and Figure 3).

### 3.5. Diagnostic Performance

**Figure 4:**
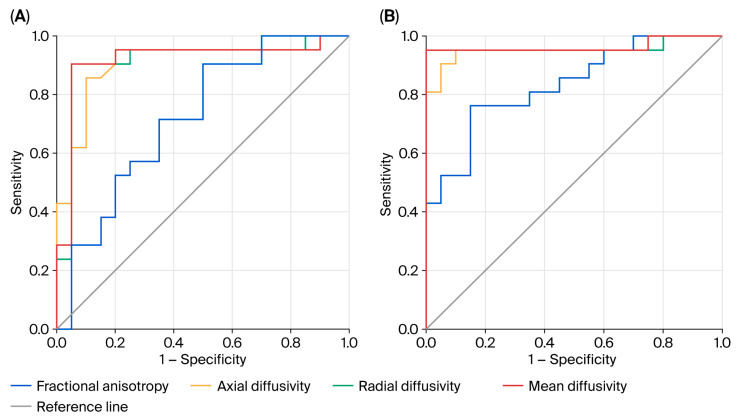
Receiver operating characteristic (ROC) curves demonstrating the diagnostic performance of diffusion tensor imaging (DTI) parameters for differentiating malignant from benign hemorrhagic brain lesions. (**A**) ROC curves based on lesion-derived DTI parameters (FA, AD, RD, and MD). (**B**) ROC curves based on lesion-to-normal (L/N) ratios of DTI parameters, showing improved discrimination compared with lesion-based metrics.

ROC analysis demonstrated excellent diagnostic performance for diffusivity-based parameters, particularly for L/N ratios (Figure 4).

### 3.6. Logistic Regression Analysis

In univariate logistic regression analysis, diffusivity-based DTI metrics were significantly associated with malignancy (all *p* < 0.001), while PEI demonstrated weaker but significant predictive value (*p* = 0.036). In multivariate models incorporating demographic variables and PEI, diffusion-derived parameters remained independently associated with malignancy across both continuous and ROC-threshold-based models, whereas age, sex, and PEI lost statistical significance after inclusion of diffusion metrics (Supplementary Tables S2–S5).

Of the 41 patients, 29 (70.7%) were scanned on the 1.5 T system and 12 (29.3%) on the 3 T system, with no association between field strength and lesion nature (χ^2^ = 0.010, *p* = 0.920). None of the absolute DTI parameters or L/N ratios differed significantly between the 1.5 T and 3 T subgroups (all *p* > 0.30; all L/N ratios *p* ≥ 0.90), and the discrimination between benign and malignant lesions remained significant within both subgroups for all L/N ratios. When magnetic field strength was added as an additional covariate to the multivariable models, the odds ratios of all diffusion parameters remained essentially unchanged and field strength was not an independent predictor of malignancy (all *p* ≥ 0.33; entered alone, OR 0.933 (0.243–3.585), *p* = 0.920). These findings indicate that magnetic field strength did not significantly affect the diffusion measurements and did not confound their diagnostic performance (Supplementary Tables S6–S8).

### 3.7. Summary of Findings

Overall, malignant hemorrhagic brain lesions were characterized by increased diffusivity and reduced anisotropy compared with benign lesions. Diffusivity-based DTI parameters—particularly L/N ratios—provided robust and independent discrimination between malignant and benign lesions, while PEI showed limited complementary value.

## 4. Discussion

The present study shows that DTI parameters—particularly AD, RD, and MD—can reliably distinguish malignant from benign brain lesions. These differences were consistent across both absolute values and L/N ratios, supporting the use of DTI-derived metrics as practical quantitative adjuncts to conventional MRI.

DTI is an advanced diffusion-based MRI technique that characterizes both the magnitude and directionality of water diffusion in biological tissues, thereby providing indirect but quantitative information about white-matter microstructure. FA reflects fiber coherence and axonal organization, whereas diffusivity-based parameters—including AD, RD, and MD—are sensitive to extracellular space expansion, myelin disruption, and axonal injury. Compared with conventional diffusion-weighted imaging, DTI allows a more detailed assessment of microstructural integrity, particularly in white-matter-rich regions where directional diffusion properties are preserved [11,15].

In hemorrhagic lesions, diffusion measurements from the lesion core are often unreliable. Blood products, susceptibility effects, and evolving hemoglobin breakdown can distort diffusion signals, resulting in artifactual alterations of both anisotropy and diffusivity metrics [9,16]. These confounding factors limit the diagnostic value of intralesional diffusion analysis and support focusing on the diagnostically informative perilesional region. In malignant lesions, this area may contain infiltrating tumor cells, reactive gliosis, vasogenic edema, microvascular remodeling, and inflammatory changes extending beyond the visible lesion margin [10,17]. These processes progressively disrupt white-matter microarchitecture, even in regions that may appear normal on conventional MRI. In contrast, benign non-neoplastic hemorrhages are more often surrounded by structurally preserved parenchyma, and perihematomal edema in these cases predominantly reflects transient plasma leakage and inflammatory response rather than true tissue infiltration [6,17]. Accordingly, diffusion alterations within the perilesional region are expected to differ fundamentally between malignant and benign lesions.

The marked increase in AD, RD, and MD observed in malignant lesions likely reflects a combination of extracellular volume expansion, tissue rarefaction, and microstructural disorganization associated with infiltrative tumor growth and necrosis. Conversely, lower FA values are consistent with loss of coherent directional diffusion barriers due to axonal injury and fiber disarray. Similar diffusion patterns—characterized by increased diffusivity and reduced anisotropy—have been reported not only within infiltrative malignant tumors but also in the surrounding peritumoral regions, reflecting tumoral infiltration [10,16]. Previous DTI studies in acute non-neoplastic intracerebral hemorrhage have also demonstrated decreased FA and increased MD, AD, and RD values within perihematomal edema regions, supporting the concept that the perilesional zone exhibits measurable microstructural diffusion alterations [18].

Receiver operating characteristic analysis supported the strong diagnostic utility of diffusivity-based DTI indices, particularly when normalized as L/N ratios. These findings indicate that diffusion-derived metrics may complement routine visual assessment in clinically ambiguous hemorrhagic lesions, where conventional morphologic cues can be masked by blood products. Logistic regression analyses further underscored the independent predictive value of diffusivity parameters after adjustment for potential confounders, highlighting the robustness of these metrics across analytic frameworks. Nevertheless, the markedly elevated odds ratios observed in ROC threshold-based models should be interpreted cautiously, as they may partly reflect limited sample size and strong group separation rather than true clinical effect magnitude.

Although the PEI also demonstrated statistical significance, its diagnostic performance was notably lower, indicating that perilesional edema alone is insufficient as a standalone discriminator. The relatively inferior performance of PEI is biologically plausible. Perilesional edema represents a heterogeneous mixture of vasogenic fluid accumulation, inflammatory changes, reactive gliosis, and—depending on the underlying etiology—variable degrees of tumor infiltration [10,17]. Because edema extent does not specifically reflect microstructural tissue disruption, indices based solely on edema volume or thickness may fail to capture the biological aggressiveness of malignant lesions. In contrast, diffusion-derived parameters directly interrogate tissue microstructure and are therefore more sensitive to subtle pathological alterations beyond the lesion margins. Consistent with these observations, previous studies focusing on perilesional diffusion characteristics have shown that diffusion metrics obtained outside the lesion core better reflect tumor-related microstructural changes than intratumoral measurements. Infiltrative high-grade gliomas demonstrate greater increases in perilesional MD and RD and more pronounced reductions in FA compared with lower-grade or non-infiltrative lesions [10,15]. Importantly, diffusion abnormalities within the perilesional zone may precede overt morphologic changes on conventional imaging, supporting the role of DTI as a sensitive marker in diagnostically equivocal cases. This is particularly relevant in hemorrhagic lesions, where acute-to-subacute blood products may obscure enhancement patterns and internal architecture, leading to misinterpretation of neoplastic hemorrhages as primary spontaneous intracerebral hemorrhage on initial imaging [1,9,19]. Recent work has further suggested that quantitative perilesional diffusion metrics may improve discrimination between high-grade gliomas and solitary brain metastases by reflecting differences in infiltrative growth patterns and the peritumoral microenvironment [20]. In the present study, malignant lesions were primarily analyzed as a single group in line with our main objective of differentiating benign from malignant hemorrhagic lesions. Nevertheless, an exploratory three-group comparison (benign, metastatic, primary malignant) showed that diffusivity-based metrics (AD/RD/MD and corresponding L/N ratios) remained higher in both malignant subgroups compared with benign lesions, while no statistically significant differences were detected between metastatic and primary malignant tumors—most likely reflecting limited statistical power due to the small metastatic sample size (*n* = 4). These findings should therefore be interpreted as hypothesis-generating.

In this context, the present findings align with a growing body of literature supporting the diagnostic utility of DTI in neuro-oncologic imaging. Several studies have demonstrated that perilesional DTI can differentiate neoplastic lesions from inflammatory, infectious, and tumor-mimicking processes by revealing increased diffusivity and reduced fractional anisotropy, reflecting greater microstructural disorganization and infiltrative behavior [5,21,22]. These observations indicate that diffusion-based microstructural alterations extend beyond specific tumor entities and may remain informative across diverse pathological conditions. In contrast to earlier work that primarily focused on glioma grading, tumor type classification, and predominantly non-hemorrhagic solid lesions, the present study extends these observations to hemorrhagic brain lesions and highlights the potential diagnostic value of perilesional microstructural assessment in clinically ambiguous scenarios.

Several limitations of this study should be acknowledged. First, the retrospective design may introduce selection bias and result in limited control over imaging protocols. MRI was not routinely performed in all patients with intracerebral hemorrhage during the study period, potentially introducing a degree of selection bias toward patients with clinically or radiologically suspected underlying lesions. Second, the relatively small sample size may restrict the generalizability of the findings. Third, all imaging examinations were performed using either 1.5 T or 3 T MRI systems depending on scanner availability and clinical workflow. Although additional analyses demonstrated no significant effect of magnetic field strength on diffusion measurements or their diagnostic performance, the use of multiple MRI systems in a retrospective study may still introduce residual scanner-related variability that cannot be completely excluded. Standardization of acquisition protocols and multicenter validation are therefore warranted. Fourth, histopathological confirmation was not available for all benign hemorrhagic lesions, and diagnoses relied on clinico-radiological follow-up (typically within 3–6 months), which may misclassify atypical or slowly evolving neoplastic cases. Fifth, DTI parameters and ROI-based measurement strategies are inherently observer-dependent; although standardized placement criteria were applied and measurements were averaged across multiple ROIs, some degree of variability in ROI selection cannot be fully excluded, particularly given the microstructural heterogeneity of the perilesional edema zone. This may contribute to spatial variability in DTI metrics across different ROI locations. Interobserver reproducibility analysis demonstrated moderate-to-good agreement for most lesion-based diffusion metrics, particularly for the L/N diffusivity ratios that also yielded the highest diagnostic performance. Lower agreement values observed in contralateral normal tissue may be related to the relatively limited variability of normal tissue measurements, where even small absolute differences between observers can proportionally reduce ICC values. In addition, subtle anatomical asymmetries between hemispheres and unavoidable variations in ROI placement may further contribute to reduced agreement in control ROI measurements. Because these values serve as the denominator for L/N ratios, variability in normal-tissue measurements may theoretically influence the reproducibility and generalizability of ratio-based metrics. However, lower agreement in the denominator does not necessarily result in a comparable reduction in the agreement of the corresponding ratios. A possible explanation is that normal-appearing white matter values exhibit a relatively narrow physiological range, making ICC estimates particularly sensitive to small measurement differences between observers, whereas lesion-side diffusion measurements show substantially greater between-subject variability. As a result, the overall variance of ratio-based metrics may be driven predominantly by lesion-side measurements rather than by small fluctuations in control ROI values. Consistent with this interpretation, the L/N diffusivity ratios that demonstrated the highest diagnostic performance in the present study also showed moderate-to-good interobserver agreement, supporting the overall reliability of these ratio-based metrics. Considering the complex spatial distribution of perilesional edema, perfect agreement would not be expected in ROI-based measurements. Future studies using volumetric or semi-automated segmentation methods may further improve measurement standardization and reproducibility. Finally, cohort heterogeneity should be acknowledged. The malignant cohort was heterogeneous (primary tumors and solitary metastases), and the small number of metastatic cases (*n* = 4) limited powered subgroup comparisons; larger cohorts are needed to validate subtype-specific effects. Nevertheless, despite histopathological heterogeneity within the malignant cohort, hemorrhagic malignant lesions may still share common biologically aggressive features, including higher proliferative activity, fragile tumor neovascularity, blood–brain barrier disruption, and extracellular microstructural disorganization, which may contribute to consistent perilesional diffusion alterations across multiple DTI parameters [23]. Recent evidence has also shown that gliomas with intratumoral hemorrhage may demonstrate higher Ki-67 indices and poorer clinical outcomes despite diverse molecular and histopathological profiles [24]. Although the benign cohort comprised heterogeneous non-neoplastic etiologies with different mechanisms of hemorrhage and edema formation, the primary objective of this study was not to differentiate among individual benign entities but rather to distinguish hemorrhagic malignant lesions from non-neoplastic causes of intracerebral hemorrhage. In routine clinical practice, radiologists are typically confronted with the broader diagnostic question of whether a hemorrhagic lesion represents an underlying malignant process or a benign condition. Similar DTI studies have likewise evaluated biologically heterogeneous neoplastic and non-neoplastic lesions as broader diagnostic categories when addressing clinically relevant differential diagnoses. For example, previous DTI investigations have grouped multiple neoplastic subtypes and diverse non-neoplastic lesions into unified comparison cohorts while still demonstrating meaningful differences in diffusion characteristics between these broader diagnostic categories [22,25]. Therefore, analyzing diverse benign hemorrhagic lesions as a single comparison group was considered both clinically relevant and reflective of real-world diagnostic practice. However, differences among benign pathologies may still have influenced diffusion measurements because different non-neoplastic pathologies can exhibit distinct perilesional microstructural characteristics. Despite these limitations, significant differences between benign and malignant lesions remained detectable across multiple diffusion parameters.

In conclusion, perilesional DTI-derived diffusivity parameters serve as objective, non-invasive biomarkers for distinguishing malignant from benign causes of intracerebral hemorrhage. The high diagnostic accuracy and encouraging reproducibility of these metrics underscore their potential role in neuro-oncologic imaging, particularly in cases where conventional imaging is inconclusive. Nonetheless, validation in larger, multi-center cohorts and incorporation into multiparametric imaging frameworks remain essential before routine clinical implementation.

## Figures and Tables

**Table 1:** DTI Acquisition Parameters.

Parameter	1.5 T MRI	3 T MRI
Sequence type	Single-shot EPI	Single-shot EPI
Diffusion directions	30	30
b-values (s/mm^2^)	0, 1000	0, 1000
TR (ms)	3248	3100
TE (ms)	94	95
Field of view (mm)	230	220
In-plane voxel size (mm^2^)	1.8 × 1.8	1.8 × 1.8
Slice thickness (mm)	5.0	5.0

**Table 2:** Interobserver Agreement (Intraclass Correlation Coefficients) for DTI Parameters.

Parameter	ICC	95% CI	Agreement	*p*-Value
**Absolute lesion values**				
Fractional anisotropy (FA)	0.612	0.38–0.77	Moderate	<0.001
Axial diffusivity (AD)	0.745	0.52–0.86	Moderate	<0.001
Radial diffusivity (RD)	0.746	0.57–0.86	Moderate	<0.001
Mean diffusivity (MD)	0.808	0.67–0.89	Good	<0.001
**Normal parenchymal values**				
Fractional anisotropy (FA)	0.653	0.44–0.80	Moderate	<0.001
Axial diffusivity (AD)	0.500	0.24–0.70	Moderate	<0.001
Radial diffusivity (RD)	0.434	0.14–0.65	Poor	<0.001
Mean diffusivity (MD)	0.482	0.22–0.68	Poor	<0.001
**Lesion-to-normal (L/N) ratios**				
FA lesion-to-normal ratio	0.654	0.43–0.80	Moderate	<0.001
AD lesion-to-normal ratio	0.791	0.64–0.88	Good	<0.001
RD lesion-to-normal ratio	0.678	0.39–0.83	Moderate	<0.001
MD lesion-to-normal ratio	0.790	0.59–0.89	Good	<0.001

ICC: intraclass correlation coefficient; CI: confidence interval; FA: fractional anisotropy; AD: axial diffusivity; RD: radial diffusivity; MD: mean diffusivity; L/N: lesion-to-normal. ICCs were estimated using a two-way random-effects model for single measurements based on absolute agreement [ICC (2, 1)] and interpreted according to Koo and Li (poor <0.50; moderate 0.50–0.75; good 0.75–0.90; excellent >0.90). All coefficients were statistically significant (*p* < 0.001).

**Table 3:** Demographic, Clinical, and Imaging Characteristics.

	Benign (*n* = 20)	Malignant (*n* = 21)	*p*-Value
**Age (Years, Mean ± SD)**	58.8 ± 20.9	63.8 ± 12.2	0.368 ^A^
**Gender**			0.873 ^B^
Male	11 (55.0%)	10 (47.6%)	
Female	9 (45.0%)	11 (52.4%)	
**Localization**			>0.999 ^B^
Right	12 (60.0%)	13 (61.9%)	
Left	8 (40.0%)	8 (38.1%)	
**MRI field strength**			0.920 ^B^
1.5 T	14 (70.0%)	15 (71.4%)	
3 T	6 (30.0%)	6 (28.6%)	
**Perilesional edema**			0.646 ^C^
0	2 (10.0%)	1 (4.8%)	
1	4 (20.0%)	10 (47.6%)	
2	5 (25.0%)	-	
3	8 (40.0%)	2 (9.5%)	
4	1 (5.0%)	8 (38.1%)	
**Tumor diameter (mm, mean ± SD)**	3.24 ± 1.21	3.38 ± 1.32	0.733 ^A^
**Overall lesion diameter** **(mm, mean ± SD)**	4.78 ± 1.96	5.97 ± 1.80	0.051 ^A^
**Perilesional Edema Index** **(PEI, median (IQR))**	34.6 (21.5–71.0)	80.6 (37.5–160.2)	0.023 ^C^

^A^ Independent samples *t*-test; ^B^ Continuity corrected χ^2^ test; ^C^ Mann–Whitney U test. IQR: Interquartile Range.

## Data Availability

The data presented in this study are available on request from the corresponding author. The data are not publicly available due to ethical reasons.

## Supplementary Materials

The following supporting information can be downloaded at https://www.mdpi.com/article/10.3390/diagnostics16132054/s1, Supplementary Table S1: Anatomical and diagnostic distribution of lesions; Supplementary Table S2: Univariate logistic regression analysis for predictors of malignancy; Supplementary Table S3: Multivariate logistic regression models (continuous variables); Supplementary Table S4: Multivariate logistic regression models using ROC-derived cut-offs for absolute lesion values; Supplementary Table S5: Multivariate logistic regression models based on lesion-to-normal ratios (L/N models). Supplementary Table S6: Comparison of DTI parameters between the 1.5 T and 3 T subgroups (all patients combined). Values are median (IQR). Supplementary Table S7: Sensitivity analysis: benign vs. malignant L/N ratios within each scanner subgroup (median; Mann–Whitney U). Supplementary Table S8: Multivariable logistic regression for malignancy: odds ratios adjusted for age, sex, and PEI, and after the further addition of magnetic field strength as a covariate.
