# Supplementary material for: Perilesional Diffusion Tensor Imaging for Differentiating Malignant and Benign Causes of Intracerebral Hemorrhage
Source: Diagnostics (Basel). 2026 Jun 30;16(13):2054. doi: 10.3390/diagnostics16132054 (PMC13359839; doi:10.3390/diagnostics16132054)
Supplement: Supplementary file 1 [file diagnostics-16-02054-s001.zip › diagnostics-4257368-supplementary.pdf]

Supplementary Table S1. Anatomical and Diagnostic Distribution of Lesions

	Benign (n=20)	Malign (n=21)
**Localization**		
Frontal	4 (20.0%)	10 (47.6%)
Parietal	4 (20.0%)	4 (19.0%)
Temporal	5 (25.0%)	3 (14.3%)
Frontoparietal	2 (10.0%)	1 (4.8%)
Frontotemporal	1 (5.0%)	1 (4.8%)
Occipital	1 (5.0%)	1 (4.8%)
Thalamus	-	1 (4.8%)
BG	1 (5.0%)	-
Insular	1 (5.0%)	-
Cerebellar	1 (5.0%)	-
**Diagnosis**		
Primary HGG	-	17 (81.0%)
Metastasis	-	4 (19.0%)
Venous ischemia	9 (45.0%)	-
Cavernoma	4 (20.0%)	-
Arterial ischemia	3 (15.0%)	-
Hematoma	2 (10.0%)	-
Hypertensive bleeding	1 (5.0%)	-
Infarct	1 (5.0%)	-

Supplementary Table S2. Univariate Logistic Regression Analysis for Predictors of Malignancy

	Odds Ratio	95% Confidence Interval		Wald	p-value
		LL	UL		
**Age**	1.018	0.981	1.057	0.865	0.352
**Female factor**	1.344	0.394	4.593	0.223	0.637
**Right lateralization**	1.083	0.309	3.802	0.016	0.901
**Absolute lesion values**					
FA *	1.111	1.017	1.214	5.419	**0.020**
FA<240	9.500	1.735	52.021	6.734	**0.009**
AD **	2.053	1.349	3.124	11.265	**<0.001**
AD>1627	54.000	8.038	362.755	16.848	**<0.001**
RD **	2.065	1.367	3.121	11.857	**<0.001**
RD>1221	180.500	15.067	2162.424	16.817	**<0.001**
MD **	2.169	1.393	3.377	11.753	**<0.001**
MD>1321	180.500	15.067	2162.424	16.817	**<0.001**
**Lesion to normal ratio**					
FA ***	1.885	1.266	2.807	9.742	**0.002**
FA<0.545	18.133	3.713	88.551	12.827	**<0.001**
AD ****	3.338	1.464	7.613	8.212	**0.004**
AD>1.378	180.500	15.067	2162.424	16.817	**<0.001**
RD ****	2.081	1.299	3.334	9.294	**0.002**
RD>1.891	n/a	n/a	n/a	n/a	n/a
MD ****	2.644	1.422	4.914	9.447	**0.002**
MD>1.654	n/a	n/a	n/a	n/a	n/a
**Perilesional edema**	1.114	0.697	1.782	0.205	0.651
**Perilesional Edema Index**	1.014	1.001	1.027	4.404	**0.036**

FA: Fractional Anisotropy, AD: Axial Diffusivity, RD: Radial Diffusivity, MD: Mean Diffusivity. LL: Lower limit, UL: Upper Limit. The impact of * corresponds to a 10-unit decrease; ** to a 100-unit increase; *** to a 0.1-unit decrease; and **** to a 0.1-unit increase in the respective parameter on malignancy. n/a: Not applicable.

Supplementary Table S3. Multivariate Logistic Regression Models (Continuous Variables)

	Odds Ratio	95% Confidence Interval		Wald	p-value
		LL	UL		
**Model 1**					
Age	0.994	0.944	1.047	0.048	0.827
Female factor	2.421	0.502	11.673	1.214	0.270
PEI (%)	1.014	1.000	1.028	3.586	0.058
FA *	1.133	1.019	1.259	5.365	**0.021**
**Model 2**					
Age	1.031	0.970	1.096	0.973	0.324
Female factor	1.254	0.199	7.889	0.058	0.810
PEI (%)	0.999	0.984	1.014	0.018	0.895
AD **	2.035	1.300	3.185	9.661	**0.002**
**Model 3**					
Age	1.007	0.946	1.073	0.053	0.818
Female factor	1.686	0.265	10.744	0.306	0.580
PEI (%)	1.004	0.989	1.019	0.249	0.618
RD **	1.946	1.287	2.945	9.944	**0.002**
**Model 4**					
Age	1.014	0.951	1.081	0.179	0.672
Female factor	1.452	0.219	9.627	0.149	0.699
PEI (%)	1.001	0.986	1.016	0.024	0.877
MD **	2.085	1.311	3.316	9.624	**0.002**

FA: Fractional Anisotropy, AD: Axial Diffusivity, RD: Radial Diffusivity, MD: Mean Diffusivity. PEI: Perilesional Edema Index. LL: Lower limit, UL: Upper Limit. The impact of * corresponds to a 10-unit decrease; ** to a 100-unit increase in the respective parameter on malignancy.

Supplementary Table S4. Multivariate Logistic Regression Models Using ROC-Derived Cut-Offs for Absolute Lesion Values

	Odds Ratio	95% Confidence Interval		Wald	p-value
		LL	UL		
**Model 1**					
Age	1.000	0.952	1.051	0.000	0.989
Female factor	1.942	0.431	8.753	0.746	0.388
PEI (%)	1.011	0.997	1.025	2.423	0.120
FA<240	8.171	1.169	57.131	4.482	**0.034**
**Model 2**					
Age	1.012	0.956	1.072	0.172	0.678
Female factor	0.807	0.116	5.618	0.047	0.829
PEI (%)	1.000	0.982	1.019	0.001	0.981
AD>1627	53.524	5.770	496.521	12.265	**<0.001**
**Model 3**					
Age	1.018	0.946	1.096	0.225	0.635
Female factor	0.586	0.047	7.368	0.172	0.679
PEI (%)	1.000	0.977	1.023	0.000	0.994
RD>1221	199.328	10.999	3612.194	12.832	**<0.001**
**Model 4**					
Age	1.018	0.946	1.096	0.225	0.635
Female factor	0.586	0.047	7.368	0.172	0.679
PEI (%)	1.000	0.977	1.023	0.000	0.994
MD>1321	199.328	10.999	3612.194	12.832	**<0.001**

FA: Fractional Anisotropy, AD: Axial Diffusivity, RD: Radial Diffusivity, MD: Mean Diffusivity. PEI: Perilesional Edema Index. LL: Lower limit, UL: Upper Limit.

Supplementary Table S5. Multivariate Logistic Regression Models Based on Lesion-to-Normal Ratios (L/N Models)

	Odds Ratio	95% Confidence Interval		Wald	p-value
		LL	UL		
**Model 1**					
Age	1.030	0.972	1.092	0.993	0.319
Female factor	3.102	0.517	18.594	1.535	0.215
PEI (%)	1.014	0.999	1.029	3.313	0.069
FA *	2.125	1.295	3.487	8.899	**0.003**
**Model 2**					
Age	1.026	0.965	1.090	0.682	0.409
Female factor	2.880	0.393	21.084	1.084	0.298
PEI (%)	1.020	1.002	1.039	4.870	**0.027**
FA<0.545	39.866	4.650	341.760	11.303	**<0.001**
**Model 3**					
Age	1.028	0.947	1.116	0.433	0.510
Female factor	7.297	0.429	124.152	1.889	0.169
PEI (%)	0.993	0.965	1.022	0.215	0.643
AD **	3.950	1.470	10.616	7.419	**0.006**
**Model 4**					
Age	1.032	0.951	1.119	0.561	0.454
Female factor	2.189	0.163	29.394	0.349	0.555
PEI (%)	1.008	0.984	1.033	0.454	0.500
AD>1.378	180.835	12.040	2715.962	14.138	**<0.001**
**Model 5**					
Age	1.093	0.947	1.261	1.464	0.226
Female factor	4.141	0.160	106.987	0.734	0.392
PEI (%)	0.997	0.970	1.024	0.058	0.809
RD **	2.203	1.246	3.894	7.376	**0.007**
**Model 6**					
Age	1.068	0.931	1.224	0.878	0.349
Female factor	7.695	0.202	292.999	1.208	0.272
PEI (%)	0.993	0.963	1.025	0.178	0.673
MD **	2.832	1.351	5.938	7.594	**0.006**

FA: Fractional Anisotropy, AD: Axial Diffusivity, RD: Radial Diffusivity, MD: Mean Diffusivity. PEI: Perilesional Edema Index. LL: Lower limit, UL: Upper Limit. The impact of * corresponds to a 0.1-unit decrease; ** to a 0.1-unit increase in the respective parameter on malignancy.

Supplementary Table S6. Comparison of DTI parameters between the 1.5T and 3T subgroups (all patients combined). Values are median (IQR).

Parameter	1.5T (n=29)	3T (n=12)	p *
FA	210 (125–262)	162 (132–180)	0.342
AD	1608 (1380–1798)	1647 (1346–1922)	0.621
RD	1199 (914–1464)	1361 (933–1574)	0.342
MD	1300 (1034–1588)	1456 (1081–1706)	0.342
FA L/N	0.597 (0.380–0.711)	0.545 (0.350–0.803)	0.899
AD L/N	1.370 (1.213–1.792)	1.394 (1.236–1.735)	0.989
RD L/N	1.848 (1.472–2.385)	1.888 (1.293–2.374)	0.899
MD L/N	1.577 (1.355–2.077)	1.653 (1.267–2.039)	0.966

** Mann–Whitney U test. AD, RD, MD in ×10^{-6} mm^2/s; FA in ×10^{-3} (as reported in the manuscript).*

Supplementary Table S7. Sensitivity analysis: benign vs. malignant L/N ratios within each scanner subgroup (median; Mann–Whitney U).

	1.5T — Benign	1.5T — Malig.	p	3T — Benign	3T — Malig.	p
FA L/N	0.697	0.405	0.008	0.808	0.348	0.015
AD L/N	1.232	1.792	<0.001	1.224	1.764	0.002
RD L/N	1.488	2.385	<0.001	1.273	2.571	0.002
MD L/N	1.357	2.077	<0.001	1.246	2.074	0.002

Supplementary Table S8. Multivariable logistic regression for malignancy: odds ratios adjusted for age, sex, and PEI, and after the further addition of magnetic field strength as a covariate.

DTI parameter	Adjusted OR (age, sex, PEI) (95% CI)	p	OR additionally adjusted for field strength (95% CI)	p	Field strength p
FA (per 10-unit decrease)	1.133 (1.019–1.259)	0.021	1.140 (1.020–1.262)	0.020	0.648
AD (per 100-unit increase)	2.084 (1.328-3.271)	0.002	2.009 (1.349–3.296)	<0.001	0.399
RD (per 100-unit increase)	2.027 (1.308-3.144)	0.002	2.009 (1.349–3.296)	0.002	0.330
MD (per 100-unit increase)	2.085 (1.311–3.316)	0.002	2.218 (1.349–3.639)	0.002	0.340
FA L/N (per 0.1 decrease)	2.125 (1.295–3.487)	0.003	2.127 (1.294–3.495)	0.003	0.902
AD L/N (per 0.1 increase)	3.950 (1.470–10.616)	0.006	3.948 (1.458–10.689)	0.007	0.993
RD L/N (per 0.1 increase)	2.203 (1.246–3.894)	0.007	2.188 (1.236–3.870)	0.007	0.772
MD L/N (per 0.1 increase)	2.832 (1.351–5.938)	0.006	2.811 (1.340–5.898)	0.006	0.857

OR, odds ratio; CI, confidence interval; PEI, perilesional edema index. Each diffusion parameter was entered in a separate model. Scaling matches the manuscript: FA per 10-unit decrease; AD/RD/MD per 100-unit increase; FA L/N per 0.1-unit decrease; AD/RD/MD L/N per 0.1-unit increase. “Field strength p” is the Wald p value for the field-strength term in the corresponding model.
